# A G-protein-coupled chemoattractant receptor recognizes lipopolysaccharide for bacterial phagocytosis

**DOI:** 10.1371/journal.pbio.2005754

**Published:** 2018-05-25

**Authors:** Miao Pan, Matthew P. Neilson, Alexander M. Grunfeld, Phillip Cruz, Xi Wen, Robert H. Insall, Tian Jin

**Affiliations:** 1 Chemotaxis Signal Section, Laboratory of Immunogenetics, National Institute of Allergy and Infectious Disease, National Institutes of Health, Rockville, Maryland, United States of America; 2 Cancer Research UK Beatson Institute, Glasgow, United Kingdom; 3 Bioinformatics and Computational Biosciences Branch, National Institute of Allergy and Infectious Disease, National Institutes of Health, Bethesda, Maryland, United States of America; Max Planck Institute for Terrestrial Microbiology, Germany

## Abstract

Phagocytes locate microorganisms via chemotaxis and then consume them using phagocytosis. *Dictyostelium* amoebas are stereotypical phagocytes that prey on diverse bacteria using both processes. However, as typical phagocytic receptors, such as complement receptors or Fcγ receptors, have not been found in *Dictyostelium*, it remains mysterious how these cells recognize bacteria. Here, we show that a single G-protein-coupled receptor (GPCR), folic acid receptor 1 (fAR1), simultaneously recognizes the chemoattractant folate and the phagocytic cue lipopolysaccharide (LPS), a major component of bacterial surfaces. Cells lacking fAR1 or its cognate G-proteins are defective in chemotaxis toward folate and phagocytosis of *Klebsiella aerogenes*. Computational simulations combined with experiments show that responses associated with chemotaxis can also promote engulfment of particles coated with chemoattractants. Finally, the extracellular Venus-Flytrap (VFT) domain of fAR1 acts as the binding site for both folate and LPS. Thus, fAR1 represents a new member of the pattern recognition receptors (PRRs) and mediates signaling from both bacterial surfaces and diffusible chemoattractants to reorganize actin for chemotaxis and phagocytosis.

## Author summary

How eukaryotic cells find and interact with bacteria is a fundamental question in biology. Eukaryotic phagocytes are cells that engulf and digest bacteria. These include single-celled organisms, such as amoeba, and cell types of multicellular organisms, such as macrophages. The current dogma is that phagocytic cells use at least two types of receptors for defense against invading pathogens: one for detecting and chasing pathogens via chemotaxis and another one for recognizing and eliminating them via phagocytosis. Detection and chasing is facilitated by G-protein-coupled receptors (GPCRs), whereas recognition and elimination employ pattern recognition receptors (PRRs). However, the social amoeba *Dictyostelium discoideum* does not encode orthologs of any known PRRs or phagocytic receptors; yet they are highly evolved as professional phagocytes that chase bacteria via chemotaxis and consume them as food through phagocytosis. Here, we show that this stereotypical phagocyte utilizes folic acid receptor 1 (fAR1), a class C GPCR, to simultaneously detect bacterial secreted folate for chasing bacteria and microbial-associated molecular patterns (MAMPs)—lipopolysaccharide (LPS)—for engulfing and consuming them.

## Introduction

How eukaryotic phagocytes locate and recognize bacteria is a fundamental question in biology. Eukaryotic phagocytes and their interactions with bacteria began when single-celled life forms, protozoans, appeared about 2 billion years ago [1]. Since then, multicellular organisms have gradually evolved increasingly complex genomes. The phagocytic cells within these organisms, such as macrophages and neutrophils, patrol the rest of the body to detect, recognize, and eliminate invading pathogenic bacteria [2,3]. The current dogma is that phagocytic cells use at least two types of receptors for defense against bacterial pathogens: one for detecting and chasing pathogens via chemotaxis and another for recognizing and eliminating them via phagocytosis. It is well established that human phagocytes locate bacteria using serpentine chemoattractant receptors linked to heterotrimeric G-proteins (hence, G-protein-coupled receptors [GPCRs]) that regulate cell shape and movement by controlling the actin cytoskeleton [4,5]. Upon catching bacteria, human phagocytes use phagocytic receptors to bind and ingest opsonized targets. Phagocytic receptors recognize opsonins, such as complements or immunoglobulins (IgGs), coated on the surface of the bacteria, and this process activates tyrosine kinases to promote actin polymerization [6–10]. In addition, infecting microorganisms are recognized by innate immune systems through pattern-recognition receptors (PRRs), such as Toll-like receptors (TLRs), scavenger receptors, and C-type lectin receptors, which collectively allow cells to recognize microbial-associated molecular patterns (MAMPs) [3,6,11,12]. However, the social amoeba *Dictyostelium discoideum*, whose protein repertoire is small compared to phagocytes from multicellular organisms, does not encode orthologs of any known PRRs or typical phagocytic receptors, such as complement receptors or Fcγ receptors [13–15]. Nonetheless, the cells are highly evolved as professional phagocytes that chase bacteria using chemotaxis and consume them as food, so they clearly contain specific receptors to mediate phagocytosis as well as chemotaxis.

*Dictyostelium* is widely used for studies of actin-linked processes such as cell migration, chemotaxis, and phagocytosis, as these processes are accomplished using a simpler set of proteins but evolutionarily conserved mechanisms [4,16–19]. Cells inhabit the soil and feed on diverse bacterial species, including gram-positive and gram-negative bacteria [20,21]. They locate bacteria by detecting metabolites such as folic acid, move toward the bacteria via chemotaxis, and then consume them through phagocytosis [22]. A previous study found that *Dictyostelium* Similar to Integrin Beta protein A (SibA) shares similar structure and function to mammalian integrin β chains and plays a role in substrate adhesion during phagocytosis [15]. However, the molecular mechanisms underlying how this phagocyte recognizes bacteria to initiate phagocytosis are not understood. We recently identified a GPCR, folic acid receptor 1 (fAR1), and demonstrated that it mediates chemotaxis toward folic acid in *Dictyostelium* [23]. Interestingly, *Dictyostelium* cells lacking fAR1 receptors (*far1*^*−*^) are defective in not only chemotaxis but also phagocytosis of *Klebsiella aerogenes* (gram-negative) [23]. The genome encodes appropriate G-proteins, including 12 Gα subunits and 1 Gβγ complex [24]. We therefore investigated the role of fAR1 and its cognate G-proteins in bacterial recognition and ingestion.

Here, we show that the stereotypical phagocyte *Dictyostelium* simultaneously utilizes fAR1 for chemotaxis and phagocytosis. The same receptor and cognate G-proteins detect the diffusible chemoattractant folate, allowing cells to locate and chase bacteria and the immobile component on the bacterial coat lipopolysaccharide (LPS) to engulf and consume them.

## Results

### fAR1 contains a Venus-Flytrap (VFT) extracellular domain and recognizes bacterial LPS

**Fig 1:**
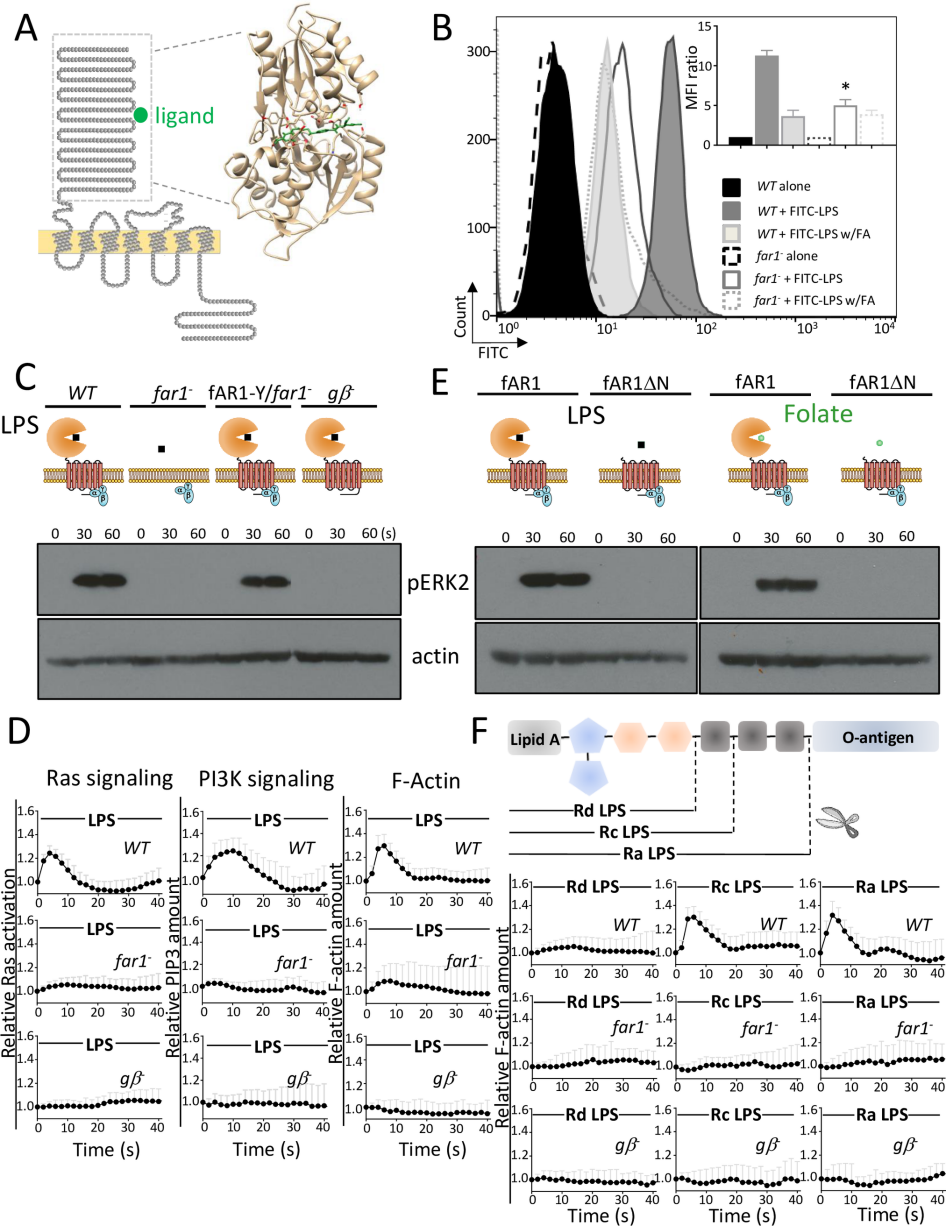
LPS triggered chemotactic signaling through fAR1. (A) fAR1 possesses a VFT domain for ligand binding. The sequence and topology of fAR1 is shown on the left. The extracellular domain of fAR1 was highlighted by a dashed box. On the right, structural modeling and computational docking predict that the extracellular domain of fAR1 folds into a VFT structure functioning as the binding site for FA moiety (green). (B) *far1*^*−*^ has decreased LPS-binding ability. The LPS binding was determined in flow cytometry by measuring the fluorescent intensity of cells binding to FITC-LPS on the surface. The representative data is shown. The MFI ratio with SD from 3 independent repetitions, which reflects the LPS binding of *WT* and *far1*^*−*^ cells in the presence or absence of FA, were graphed. A Student *t* test indicated a statistically significant difference in LPS binding between *far1*^*−*^ and *WT* cells (* indicates *P* < 0.01). (C) ERK2 signaling triggered by LPS is impaired in *far1*^*−*^ and *gβ*^*−*^ cells. ERK2 activation in vegetative *WT*, *far1*^*−*^, *gβ*^*−*^, and fAR1-Y/*far1*^*−*^ cells in response to 100 μg/ml LPS stimulation was examined. ERK2 activation was determined by immunoblotting with anti–phospho-ERK2 antibody, using actin as a loading control. (D) LPS-induced Ras activation, PIP3 signaling, and actin polymerization are mainly dependent on fAR1 and Gβ. Vegetative *WT* and mutant cells expressing RBD-GFP, PH_CRAC_-GFP, and LimEΔcoil-GFP were stimulated with 100 μg/ml LPS at 0 s. The transient increase in fluorescence intensity was measured at the plasma membrane and graphed. The intensity of the GFP signal was normalized to the first frame of each set of cells. Mean and SD from 10 cells are shown for the time course. A Student *t* test indicated a statistically significant difference in fluorescence intensity peak value between *far1*^*−*^, *gβ*^*−*^, and *WT* cells (*P* < 0.01). (E) VFT domain of fAR1 is essential for ERK2 activation by LPS and FA. ERK2 activation in vegetative fAR1-Y/*far1*^*−*^ and fAR1ΔN-Y/*far1*^*−*^ cells in response to 100 μg/ml LPS or 100 μM FA stimulation was examined by immunoblotting with anti–phospho-ERK2 antibody, using actin as a loading control. (F) fAR1 recognizes saccharide region in LPS to transduce signal. Schematic structure of bacterial LPS molecule, which contains lipid A, core region, and O-antigen. Mutant LPS molecules are composed of same lipid A but different saccharides in core region. Vegetative *WT*, *far1*^*−*^, and *gβ*^*−*^ cells expressing LimEΔcoil-GFP were stimulated with 100 μg/ml different LPS at 0 s. The transient increase in fluorescence intensity was measured at the plasma membrane and graphed. The intensity of the GFP signal was normalized to the first frame of each set of cells. Mean and SD from 10 cells are shown for the time course. A Student *t* test indicated a statistically significant difference in fluorescence intensity peak value between *far1*^*−*^, *gβ*^*−*^, and *WT* cells triggered by Ra- and Rc-LPS (*P* < 0.01). There is no significant difference in fluorescence intensity peak value between mutants and *WT* cells triggered by Rd-LPS under the test condition. Underlying data can be found in S1 Data. ERK2, extracellular signal-regulated kinase 2; FITC, fluorescein isothiocyanate; FA, folic acid; fAR1, folic acid receptor 1; GFP, green fluorescent protein; LimEΔcoil, partial sequences of LimE protein; LPS, lipopolysaccharide; MFI, mean fluorescence intensity; PH_CRAC_, PH domain of cytosolic regulator of adenylyl cyclase; PIP3, phosphatidylinositol (3,4,5)-trisphosphate; RBD, Ras binding domain; VFT, Venus-Flytrap; WT, wild-type.

To explore how fAR1 recognizes bacteria, we first analyzed its amino acid sequence as detailed in Materials and methods. We found that fAR1 contains an amino-terminal extracellular domain, seven transmembrane domains, and a carboxyl-terminal intracellular domain (Fig 1A). Structural alignment and homology modeling of the extracellular domain including approximately 350 amino acids show that it folds as a VFT structure. Computational docking analysis indicates that a folic acid molecule binds to the VFT cleft of fAR1 (Fig 1A). VFT modules are found in various membrane proteins in organisms ranging from bacteria (such as periplasmic binding proteins) to higher metazoans in which they constitute the ligand-binding domains of the class C GPCRs, including glutamate receptors (mGluRs), gamma-aminobutyric acid type B receptors (GABA_B_Rs), Ca^2+^-sensing receptors (CaSR), taste receptors (T1R), pheromone receptors (V2R), and olfactory receptors [25]. Since VFT domains originated from bacterial periplasmic binding proteins and interact with various ligands [26,27], we conjecture that the VFT domain of fAR1 can bind molecules on the bacterial surface in addition to the diffusible chemoattractant folic acid. It was recently reported that *far1*^*−*^ cells are defective in phagocytosis of *K*. *aerogenes* (gram-negative) but appear to be normal in phagocytosis of *Bacillus subtilis* (gram-positive) [23,28]. Thus, we examined whether a major MAMP in gram-negative bacterial outer membranes—LPS—binds fAR1 to promote engulfment.

Cells of wild type (*WT*) or *far1*^*−*^ were incubated with fluorescently labeled LPS at 4°C for 15 min, and fluorescent cells were quantified by flow cytometry (Fig 1B). Binding of fluorescein isothiocyanate (FITC)-LPS to *far1*^*−*^ cells was significantly reduced compared to that of *WT* cells, and excessive folic acid (1 mM) reduced binding of LPS to *WT* cells. Taken together, these data indicate that LPS binds fAR1, and the binding sites of LPS and folic acid may overlap.

We then examined fAR1/G-protein-mediated chemotactic signaling in response to LPS (Fig 1C). LPS stimulation induced extracellular signal-regulated kinase 2 (ERK2) activation by phosphorylation in *WT* but not *far1*^*−*^ or *gβ*^*−*^ cells, while expressing fAR1–yellow fluorescent protein (YFP) in *far1*^*−*^ cells restored LPS-induced ERK2 activation (Fig 1C). Cells expressing Ras binding domain (RBD)–green fluorescent protein (GFP), PH domain of cytosolic regulator of adenylyl cyclase (PH_CRAC_)-GFP, and partial sequences of LimE (ΔlimE)-GFP—which are the fluorescent probes for monitoring activation of Ras, phosphatidylinositol-4,5-bisphosphate 3-kinase (PI3K), and actin polymerization, respectively [29–31]—were stimulated with soluble LPS and imaged using time-lapse fluorescence microscopy (Fig 1D and S1 Fig). LPS, like folic acid, induced transient membrane translocations of RBD-GFP (Ras signaling), PH-GFP (PI3K signaling), and ΔLimE-GFP (F-actin) in *WT* cells, which is substantially decreased in *far1*^*−*^ or *gβ*^*−*^ cells (Fig 1D and S1A–S1D Fig). Together, our results indicate that binding of LPS to fAR1 activates heterotrimeric G-proteins that trigger chemotactic signaling events.

### The VFT domain in fAR1 recognizes folic acid and saccharides in the core region of LPS

To further explore the role of fAR1’s VFT domain in ligand recognition, we compared ligand-induced activation of ERK2 between *far1*^*−*^ cells expressing fAR1-YFP and a mutant fAR1 lacking the N-terminal VFT domain, fAR1ΔN-YFP (Fig 1E and S2A Fig). Although both fAR1-YFP and fAR1ΔN-YFP are localized at the membrane (S2B Fig), ERK2 activation by LPS or folic acid was only detected in cells expressing fAR1-YFP (Fig 1E), supporting the notion that both ligands bind to the VFT domain to activate fAR1.

To identify the crucial region of LPS recognized by fAR1, we examined chemotactic signaling induced by LPS molecules produced by different bacterial mutants, which contain the same lipid A moiety but different types of saccharide context (Fig 1F). Ra-LPS and Rc-LPS, but not Rd-LPS, triggered robust actin polymerization in *WT* cells (Fig 1F and S2C–S2E Fig). As expected, mutant LPS-mediated signaling was absent in *far1*^*−*^ and *gβ*^*−*^ cells (Fig 1F and S2C–S2E Fig), suggesting that saccharides in the core region of LPS bind to and activate fAR1.

### A chemotactic system also drives the engulfment of particles coated with chemoattractants

**Fig 2:**
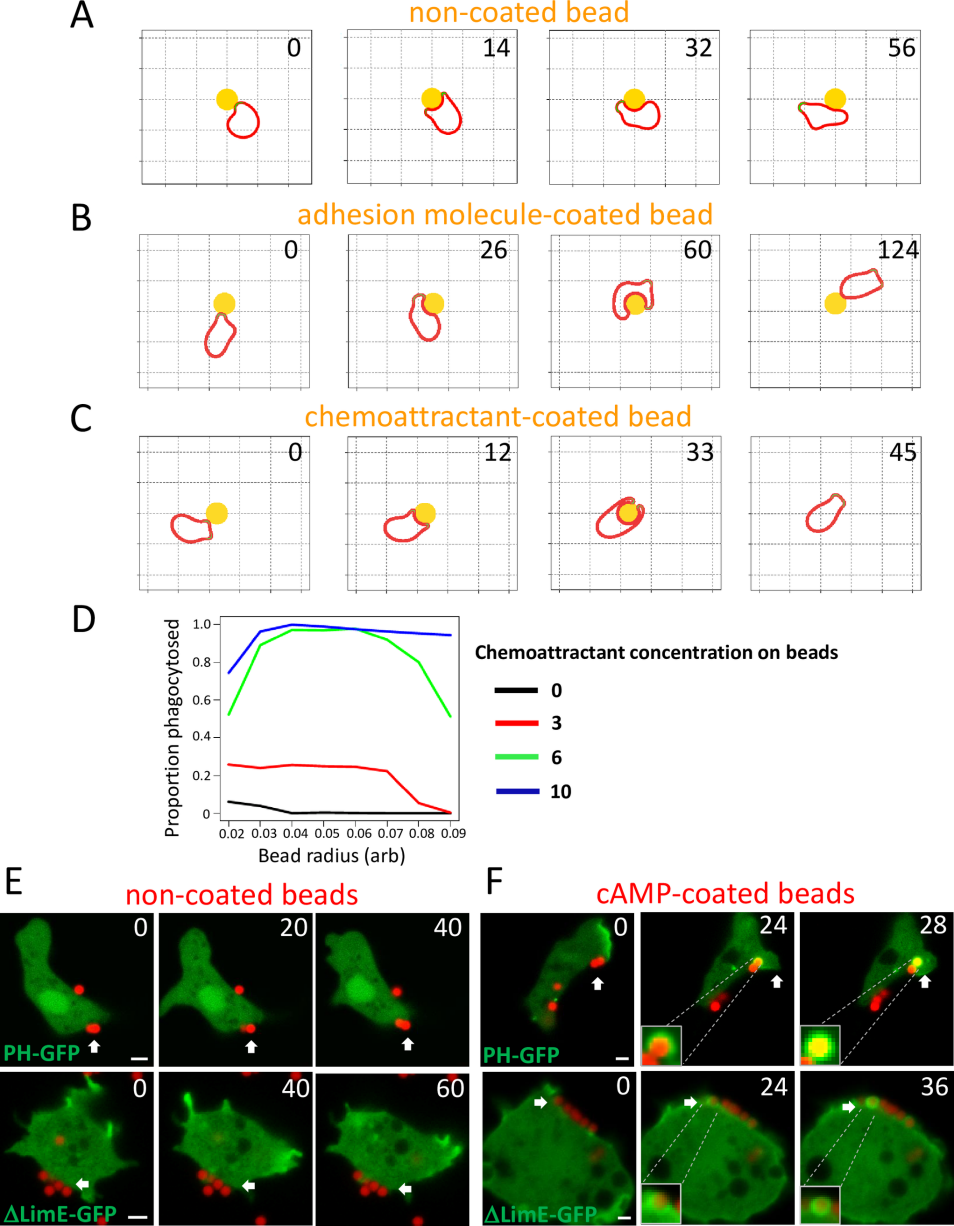
A chemoattractant-sensing machinery promotes the engulfment of particles. (A) Simulated cell migration in the presence of a circular obstacle without any coating. The cell fails to engulf the obstacle. (B) Simulation of cell migration in the presence of circular obstacle coated with adhesive molecules, which increases interaction between cell and obstacle but fails to promote engulfment. (C) Simulated cell migration in the presence of a circular obstacle coated with chemoattractant on surface, which promotes engulfment. (D) Engulfment efficiency is dependent on the concentration of chemoattractant on the surface of the obstacle. (E) Developed *D*. *discoideum WT* cells expressing PH_CRAC_-GFP and and LimEΔcoil-GFP were incubated with NeutrAvidin beads. The beads failed to trigger signaling events and engulfment. Scale bar: 2 μm. F. cAMP coated on the beads triggers PIP3 signaling and actin polymerization for engulfment in developed *D*. *discoideum* cells. Phagocytosis of 1 μm cAMP-coated beads (red) by developed *WT* expressing PH_CRAC_-GFP, LimEΔcoil-GFP (green). Scale bar, 2 μm. Underlying data can be found in S1 Data. PH_CRAC_, PH domain of cytosolic regulator of adenylyl cyclase; LimEΔcoil, partial sequences of LimE protein; PIP3, phosphatidylinositol (3,4,5)-trisphosphate; WT, wild-type

In previous work, we found a relatively simple computational model could explain how biased positive feedback of actin regulators could lead to amoeboid chemotaxis [32]. The activation of the cytoskeleton is mediated by an activator at the cell surface, which drives movement by causing pseudopod protrusion in the same way as the actin system [33,34]. Chemoattractants do not directly cause protrusion but modulate the positive feedback that maintains pseudopods. When this model was adapted to respond appropriately to rigid obstacles, by stalling parts of the pseudopod that were unable to move forward, we were surprised to see behavior that closely resembled phagocytosis—when pseudopods hit particles, they split in halves that progressed down the sides of the particle and started to surround it. However, with simple particles (Fig 2A and S1 Video), the nascent cup became unstable and resolved into a pseudopod before the particle was halfway engulfed. Modeling physical adhesion between the virtual cell and the particle (Fig 2B and S1 Video) increased the amount and duration of contact, but it still usually failed before engulfment, leading the cell to migrate away from the particle. In contrast, when the particle was treated as if it were coated with an immobilized chemoattractant, both halves of the nascent phagocytic cup were stabilized, and the cell efficiently engulfed the particle (Fig 2C and S1 Video). Increasing the chemoattractant concentration on the particle surface increased the engulfment efficiency; however, increasing the amount of adhesion failed to do so (Fig 2D and S3A Fig). This model provides a plausible mechanism for why G-protein-linked signaling can be important for phagocytic efficiency, as has been found in multiple systems [35,36], as well as its better-known role in chemotaxis.

### Chemoattractant GPCR/G-protein machineries promote engulfment of the particles coated with immobile attractants

To validate that chemoattractants on a particle surface promote phagocytosis by amoebas, we examined the engulfment of chemoattractant-coated beads. At the aggregating stage, *Dictyostelium* cells chemotax robustly to cAMP but lose the ability to eat bacteria [37]. Using live cell imaging, we observed that cAMP-coated beads—but not uncoated beads—induced localized signaling responses, such as accumulation of PH_CRAC_-GFP and ΔlimE-GFP, followed by bead engulfment (Fig 2E and 2F). Previous studies indicated that cAMP receptor 1 (cAR1) works mainly with Gα2Gβγ subunits [38–40], while fAR1 may couple with Gα4Gβγ subunits [41–43]. We found that cAMP-coated beads induced these localized responses with subsequent phagocytic cup formation in *WT*, *gα4*^*−*^, and *far1*^*−*^ cells (S3B Fig) but not in *car1*^*−*^, *gβ*^*−*^, or *gα2*^*−*^ cells, in which cAMP sensing is abolished (S3C Fig). Consistent with this, coatings of interleukin 8 (IL-8), a potent chemokine for human neutrophils, are reported to promote engulfment by neutrophils [44]. Using live cell imaging of HL60 cells, a human neutrophil cell line, we observed that beads coated with IL-8—but not uncoated beads—induced actin polymerization to form a phagocytic cup, followed by engulfment (S3D and S3E Fig). In addition, IL-8 beads failed to trigger engulfment when Gi signaling was blocked by pertussis toxin treatment, indicating that the chemokine IL-8 receptor and its heterotrimeric Gi-proteins are required for engulfment (S3F Fig). Furthermore, we previously showed that folic acid–coated beads can trigger localized chemotactic responses, leading to fAR1-mediated engulfment by *Dictyostelium* amoeba [23]. These results indicate that chemoattractants immobilized on the surface of particles activate GPCR/G protein systems to induce the formation of a phagocytic cup that leads to particle engulfment by amoeba of both *Dictyostelium* and mammals.

### LPS-induced activation of fAR1/G-proteins mediates both cell migration and particle engulfment

**Fig 3:**
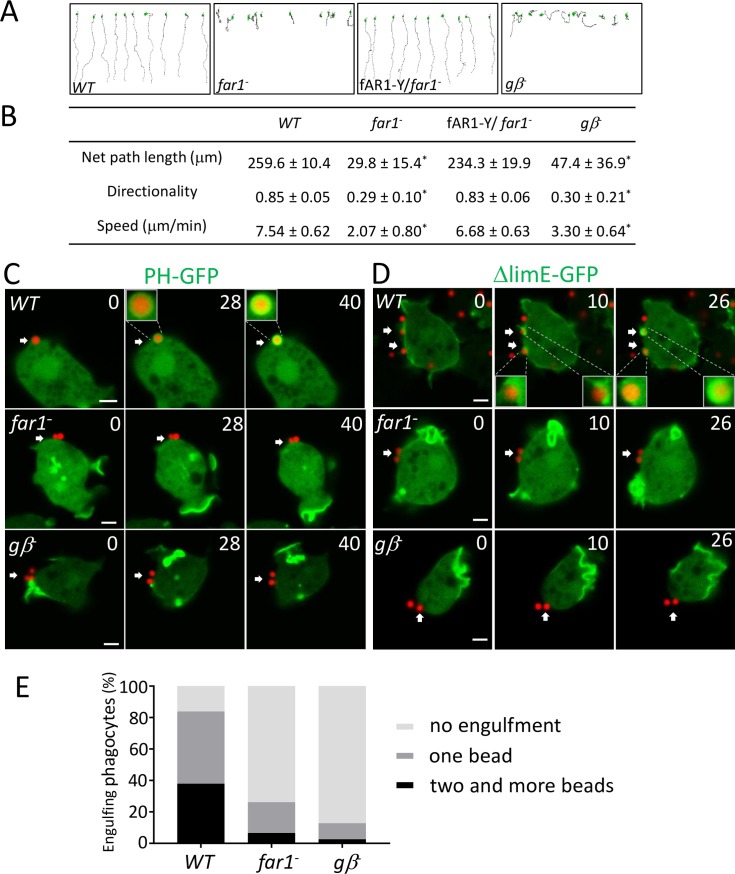
fAR1/G protein machinery mediates LPS-induced cell migration and particle engulfment. (A) EZ-TAXIScan chemotaxis toward a linear LPS gradient of vegetative *WT*, *gβ*^*−*^, *far1*^*−*^, and fAR1-Y/*far1*^*−*^ cells. Migration paths toward LPS are shown. (B) Ten cells of each strain from (A) were used for tracing. The mean and SD resulting from quantification of chemotaxis parameters are shown. A Student *t* test indicated a statistically significant difference between *gβ*^*−*^, *far1*^*−*^, and *WT* cells (* indicates *P* < 0.01). (C) LPS on particle surface triggers localized PIP3 signaling and engulfment. Engulfment of 1 μm LPS-coated beads (red) by *WT* but not *far1*^*−*^ or *gβ*^*−*^ cells expressing PH_CRAC_-GFP (green). Scale bar: 2 μm. (D) LPS on particle surface triggers localized actin polymerization to form phagocytic cup. Engulfment of 1 μm LPS-coated beads (red) by *WT* but not *far1*^*−*^ or *gβ*^*−*^ cells expressing LimEΔcoil-GFP (green). Scale bar: 2 μm. (E) LPS triggers engulfment through fAR1 and Gβ. Quantitation of engulfment movies from C and D to compare engulfment ability between *WT*, *far1*^*−*^, and *gβ*^*−*^ cells. A Student *t* test indicated a statistically significant difference in percentage of cell-engulfing LPS-beads between *far1*^*−*^, *gβ*^*−*^, and *WT* cells (*P* < 0.01). Underlying data can be found in S1 Data. fAR1, folic acid receptor 1; LimEΔcoil, partial sequences of LimE protein; LPS, lipopolysaccharide; PH_CRAC_, PH domain of cytosolic regulator of adenylyl cyclase; PIP3, phosphatidylinositol (3,4,5)-trisphosphate; WT, wild-type; LimEΔcoil, partial sequences of LimE protein

Next, we tested how LPS originated from bacterial surface influences cell movement and engulfment. To determine whether the binding of LPS to fAR1 directs cell migration, we performed the EZ-TAXIScan chemotaxis assay [45] to test the ability of *WT*, *far1*^−^, fAR1-Y/*far1*^−^, and *gβ*^*−*^ cells migrating in a linear gradient of soluble LPS (Fig 3A). We found that soluble LPS, like folic acid, functions as an attractant to guide chemotaxis for *Dictyostelium*. *WT* and fAR1-YFP/*far1*^*−*^ moved to LPS with similar net path lengths, speeds, and directionality, while *far1*^*−*^ and *gβ*^*−*^ cells did not chemotax to LPS (Fig 3A and 3B and S4 Fig). These findings suggest that fAR1 couples with heterotrimeric G-proteins to mediate chemotaxis toward LPS.

To examine whether interactions between fAR1 and surface-bound LPS promote engulfment, we incubated biotin-labeled LPS with NeutrAvidin beads to generate LPS-coated beads and then mixed them with live cells (Fig 3C and 3D and S2 Video). LPS-coated beads induced a localized phosphatidylinositol (3,4,5)-trisphosphate (PIP3) response (arrows in the upper panel of Fig 3C) and actin polymerization (arrows in the upper panel of Fig 3D) around the beads, subsequently leading to bead engulfment. To dissect the function of fAR1 and heterotrimeric G proteins in LPS-mediated engulfment, we then imaged *far1*^−^ and *gβ*^−^ cells that had been incubated with LPS-coated beads for 5 min and counted cells with or without internalized beads (Fig 3C and 3D and S2 Video). Under similar conditions, more than 80% of *WT* cells engulfed 1 or more beads, while less than 30% of *far1*^*−*^ cells or 20% of *gβ*^*−*^ cells engulfed beads (Fig 3E). Our results demonstrate that the fAR1/G-protein system detects both diffusible and immobile ligands and activates the pathways leading to either cell migration toward the source of soluble attractants or to the engulfment of a particle coated with recognition patterns.

### fAR1 coupled with heterotrimeric G-proteins mediates engulfment of *K*. *aerogenes*

**Fig 4:**
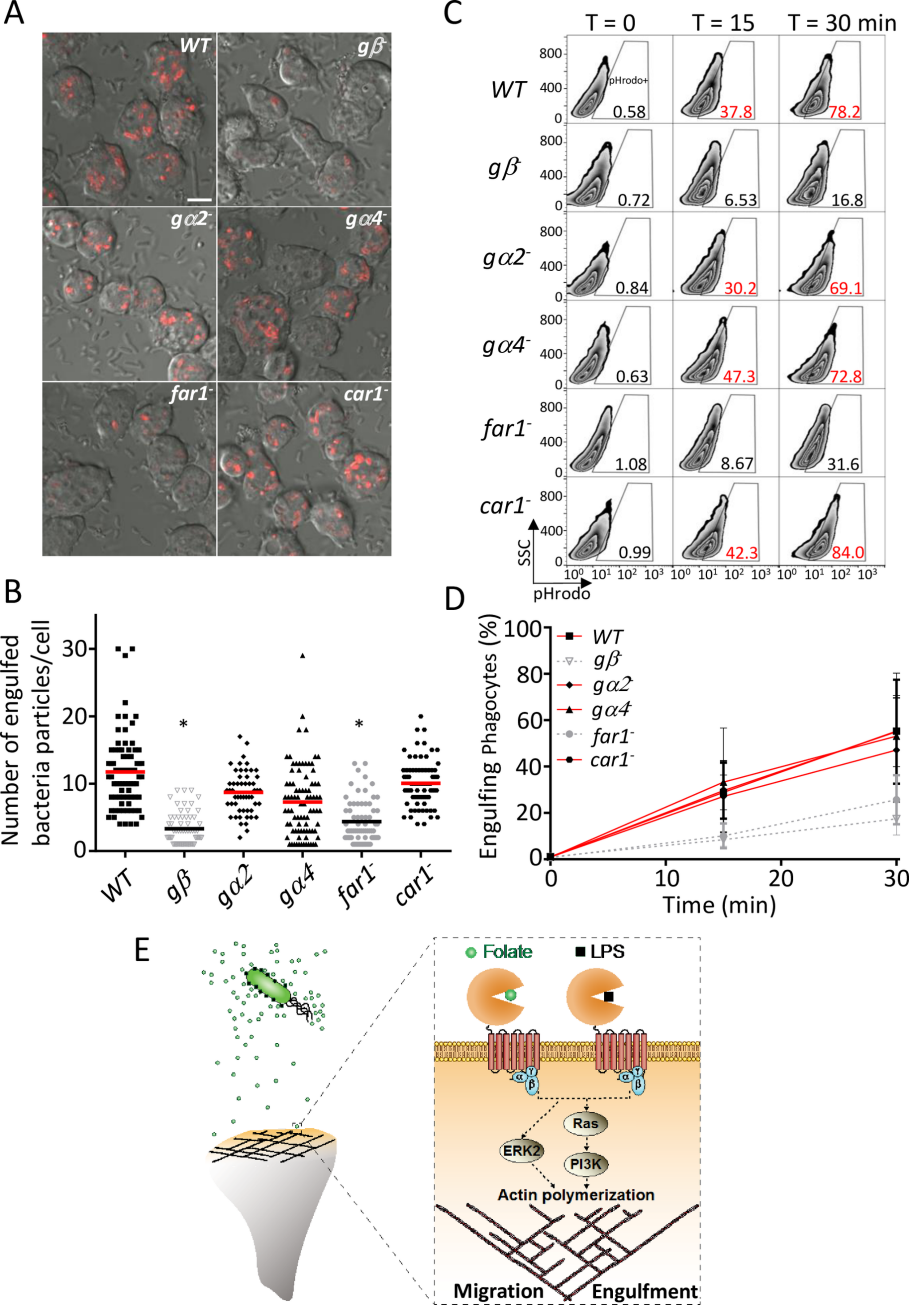
fAR1/G protein machinery mediates live *K*. *aerogenes* engulfment. (A) *WT* and mutants were mixed with pHrodo-labeled *K*. *aerogenes* at a 1:50 ratio. After 20 min, cells were mounted on a slide in basic pH buffer and analyzed by confocal microscopy. The representative data are shown. The engulfed pHrodo-labeled *K*. *aerogenes* are shown as red; Scale bars, 5 μm. (B) The engulfed bacterial number in each cell from (A) was measured and plotted for *WT* and mutant cells. A Student *t* test indicated a statistically significant difference in number of engulfed bacteria per cell between *far1*^*−*^, *gβ*^*−*^, and *WT* cells (* indicates *P* < 0.01). (C) *WT* and mutant cells were mixed with pHrodo-labeled live *K*. *aerogenes* at a 1:100 ratio for the indicated time. Cells were suspended in basic pH buffer and analyzed for the percentage of pHrodo positive cells by flow cytometry, which represents the cells that engulfed *K*. *aerogenes*. Quantification of engulfed *K*. *aerogenes* is compared between different *Dictyostelium* strains. (D) The mean and SD resulting from quantification of 3 independent repetitions of the experiments exemplified in (C) are plotted. (E) fAR1 recognizes not only diffusible chemoattractant but also immobilized ligand on bacterial surface to mediate both migration and engulfment. Underlying data can be found in S1 Data. ERK2, extracellular signal-regulated kinase 2; fAR1, folic acid receptor 1; LPS, lipopolysaccharide; PI3K, phosphatidylinositol-4,5-bisphosphate 3-kinase abbreviation SSC, side scatter; WT, wild type.

To further test the roles of GPCR/G-protein systems in bacterial engulfment, we examined engulfment of live *K*. *aerogenes* by *WT*, *gβ*^*−*^, *gα2*^*−*^, *gα4*^*−*^, *far1*^*−*^, and *car1*^*−*^ cells (Fig 4). We first imaged internalization of *K*. *aerogenes* labeled by the pHrodo fluorescence probe using confocal microscopy (Fig 4A). Cells were incubated with pHrodo-labeled *K*. *aerogenes* for 20 min, mixed with a basic buffer to quench extracellular pHrodo fluorescence, and then imaged by confocal microscopy. *WT* cells effectively engulfed the bacteria and formed acidified phagolysosomes containing internalized pHrodo-labeled *K*. *aerogenes* emitting fluorescence signals (red particles), as the low pH environment in phagolysosomes enhances the fluorescence of pHrodo. Relative to *WT* cells, *gβ*^*−*^ and *far1*^*−*^ cells displayed a significant decrease in bacterial internalization (Fig 4A and 4B). We then measured the internalization of *K*. *aerogenes* using flow cytometry (Fig 4C and 4D). Cells were incubated with pHrodo-labeled *K*. *aerogenes*, collected at the indicated time points, and analyzed by flow cytometry to quantify the pHrodo-positive cells that contained internalized bacteria (Fig 4C). Compared to *WT* cells, *gβ*^*−*^ and *far1*^*−*^ cells were substantially defective in bacterial uptake over time, while *gα2*^*−*^, *gα4*^*−*^, and *car1*^*−*^ cells still retained the ability to internalize bacteria (Fig 4D). Taken together, our results suggest that ligands on the surface of *K*. *aerogenes* activate fAR1, which links to Gβγ and 1 or more Gα subunits to mediate bacterial engulfment.

## Discussion

The evidence presented here and previously [23] reveals that the stereotypical phagocyte *D*. *discoideum* utilizes a GPCR/G-protein machinery to simultaneously detect a diffusible chemoattractant folate and recognize an immobile component, LPS, on the bacterial outer membrane for both chasing and engulfing bacteria (Fig 4E). We find that fAR1 is different from other chemoattractant GPCRs, as it belongs to the class C GPCR family and consists of a VFT extracellular domain for sensing multiple ligands. fAR1 functions as a PRR to mediate bacterial engulfment. The VFT domain in fAR1 recognizes LPS, a generic signature in commensal and pathogenic bacteria. VFT domains originated from bacterial periplasmic acid-binding proteins (PBPs), which bind amino acids, sugars, and other nutrients for bacterial growth. These domains have been acquired and adopted as extracellular ligand-binding domains by cell membrane receptors [46], such as those in the class C GPCR family. Our analysis indicates that the *D*. *discoideum* genome encodes 14 class C GPCRs, including fAR1, that contain extracellular VFT domains. Bioinformatics analysis suggested that the VFT domain of these members are evolutionarily closer to bacterial PBPs and eukaryotic GABA_B_Rs family than to other class C GPCRs [47,48], constituting the prototype of class C GPCRs with a variety of physiological functions [49]. Interestingly, bacterial PBPs bind various ligands, while most mammalian class C GPCRs expressed in the central nerve system bind only one natural ligand, implying that during evolution, VFT domains of class C GPCRs underwent partial loss of function, such as the ability to bind various ligands [49]. However, the VFT in fAR1 still retains the ability to recognize two different ligands: the bacterial-secreted diffusible chemoattractant folate for chasing bacteria and the major MAMP, LPS for engulfing gram-negative bacteria. Thus, the chemoattractant fAR1 receptor, a VFT containing GPCR, represents a new subfamily of PRRs.

It is intriguing to note that most known LPS receptors recognize the conserved lipid A moiety [50–53], while fAR1 mainly responds to the saccharide core region of LPS instead. The lipid A moiety inserts into the membrane and links to a core complex of 8–12 sugars, which is linked to the O-antigen [54]. Interestingly, previous studies indicated that *Dictyostelium* may sense saccharides to mediate engulfment [55–57]. In addition, a recent study showed that brain angiogenesis inhibitor I (BAI1), an adhesion GPCR found in mammals, also recognizes the saccharide cores of LPS and promotes the engulfment of gram-negative bacteria [58]. The saccharide cores of LPS protruding outward from the bacteria membrane may be used as a target of recognition by phagocytes that engulf live bacteria. In the meantime, subtle changes on MAMPs from pathogen may substantially prevent phagocyte detection [59,60]. We noticed that *far1*^−^ cells still maintain a low level of binding for either LPS or folic acid, suggesting that other proteins interacting with LPS and folic acid may exist on the cell surface. Future study is needed to identify them.

In conclusion, our current study on the social amoeba *D*. *discoideum* sheds new light on the origin of bacterial recognition by eukaryotic phagocytes, the path through which PRRs evolved, and the unexpectedly close mechanistic connection between chemotaxis and phagocytosis. One key question that remains is how *D*. *discoideum* recognizes gram-positive bacteria. A study reported that *Dictyostelium* cells lacking fAR1 lost the ability to chemotax toward gram-positive and gram-negative bacteria but still retained the ability to phagocytose *B*. *subtilis*, a gram-positive bacterium [28]. Thus, other receptors must be involved for recognizing the outer-membrane components of gram-positive bacteria to mediate their engulfment, and studies are now underway to determine how *Dictyostelium* cells recognize those MAMPs.

## Materials and methods

### Cell culture and development

For axenic culture, vegetative cells were grown in D3T medium at 22°C with or without antibiotics as required. For synchronous development in shaking suspension, cells were harvested at mid-log phase, washed in development buffer (DB; 7.4 mM NaH_2_PO4⋅H_2_O, 4 mM Na_2_HPO_4_⋅7H_2_O, 2 mM MgCl_2_, 0.2 mM CaCl_2_, pH 6.5) twice, and then resuspended in DB to 2 × 10^7^ cells/ml. Cells were rotated at 120 rpm on a platform shaker at 22°C for 5 h and given exogenous 75 nM pulses of cAMP every 6 min. Cells of *gα2*^*−*^ and *gα4*^*−*^ were obtained from DictyBase stock center [61]. Cells of *car1*^−^ and *gβ*^*−*^ were provided by Peter Devreotes lab.

### ERK2 activation

*WT* and different mutant *Dictyostelium* cells were grown in D3-T medium, washed twice with phosphate buffer (PB; 7.4 mM NaH_2_PO4⋅H_2_O, 4 mM Na_2_HPO_4_⋅7H_2_O, pH 6.5), and resuspended in PB at 2 × 10^7^ cells/ml. Cells were stimulated with 100 μM folic acid (Sigma) or 100 μg/ml LPS (*Escherichia coli* O111:B4, Sigma). At indicated time intervals after the stimulation, 150 μl of cell suspension was taken out, mixed with 50 μl 4× sample buffer, and boiled for 3 min. Proteins were separated by SDS–PAGE, transferred to nitrocellulose membranes, and blotted with polyclonal anti–phospho-p44/p42 MAPK (pERK2) antibody (Cell Signaling Technology) and anti-actin antibody (Santa Cruz Biotechnology).

### PH-GFP, RBD-GFP, and LimEΔcoil-GFP translocation upon LPS stimulation

Vegetative *WT* and mutant *Dictyostelium* cells expressing PH_CRAC_-GFP, RBD-GFP, or LimEΔcoil-GFP were prepared using the same protocol for ERK2 activation assay. Cells were plated in 4 well chambers (Lab-Tek) and then imaged with a Zeiss LSM 880 Laser Scanning Microscope with a 60×; 1.3 NA Plan-Neofluar objective lens. Fluorescent frames were acquired every 2 s and in 30 frames total. A final concentration of 100 μg/ml LPS purified from different *E*. *coli* strains (O111:B4, Ra, Rc, and Rd, Sigma) was added to the cells to induce RBD-GFP, PH_CRAC_-GFP, or LimEΔcoil-GFP translocation from the cytosol to the plasma membrane. The temporal–spatial intensity changes of RBD-GFP, PH_CRAC_-GFP, or LimEΔcoil-GFP in cells were directly imaged using a confocal microscope. For each cell, a region of interest (ROI) was drawn at the plasma membrane to measure the fluorescence intensity change over time. The fluorescence intensities were normalized to the first frame with the appearance of LPS stimulation, which is defined as 1.

### EZ-TAXIScan chemotaxis assay

*WT* and mutant *Dictyostelium* cells were harvested from vegetative stage, washed with PB, and resuspended at 1 × 10^6^ cells/ml. Cell migration was recorded in 15 s intervals at 22°C for 40 min in the EZ-TAXIScan chamber (as indicated in Fig 3), which was assembled as described in the manufacturer’s protocol. Chips used in the chamber were precoated with 1% BSA at 22°C for 30 min. A stable gradient of 0–1,000 μg/ml LPS, as described in Fig 3 and S4 Fig, was established for the assay. Cell migration analysis was performed with DIAS software.

### Ligand conjugated beads and non-coated beads

To make biotinylated cAMP, 50 μl of 18 mM biotin EZ-Link Sulfo-NHS-Biotin (Thermo Fisher) was incubated with 100 μl of 10 mM 6-AH-cAMP (Biolog) at 22°C for 8 h and purified by HPLC. NeutrAvidin beads with 1-μm diamter (Thermo Fisher) were washed with PB 3 times and resuspended into 1 ml PB. The beads were then incubated with biotinylated cAMP at 22°C for 2 h. The coated beads were washed 5 times with precold PB to remove excess free ligand. To make LPS-labeled beads, biotinylated bacterial LPS (*E*. *coli* O111:B4, InvivoGen) was incubated with 1 μm NeutrAvidin beads 22°C for 2 h. The coated beads were washed 5 times with precold PB to remove excess free ligand. The non-coated NeutrAvidin beads or red fluorescent beads (Thermo Fisher) were washed 5 times with PB before use.

### Bacterial engulfment assays

Bacteria engulfment by *Dictyostelium* was conducted in both suspension and adhesion cultures as previously described [23,41]. Overnight cultured *K*. *aerogenes* were labeled with pHrodo Red dye (Life Technology) and incubated with axenic *Dictyostelium* cells in phosphate buffer at a 100:1 ratio at 22°C in suspension cultures (150 rpm). At indicated times, the cells were centrifuged and resuspended in basic buffer (50 mM Tris pH 8.8 and 150 mM NaCl) to quench the fluorescence of nonphagocytized pHrodo-labeled *K*. *aerogenes*. The phagocytes and *K*. *aerogenes* were distinguished by forward scatter (FSC) and side scatter (SSC). The appearance of pHrodo in the phagocyte population was monitored as an indicator of *K*. *aerogenes* engulfment. The phagocyte cell population characterized by high fluorescence of pHrodo was considered as the cells that engulfed *K*. *aerogenes*. Data acquisition and analysis were done using FACSort flow cytometer (BD Bioscience) with Cell Quest software (v. 3.3) and analyzed using FlowJo (v. 10.0.8; Tree Star). Quantification of engulfed bacteria number per *Dictyostelium* cell was analyzed using confocal microscopy. *Dictyostelium* cells were allowed to attach onto 4 well chambers (Lab-Tek) and then incubated with pHrodo labeled *K*. *aerogenes* in phosphate buffer. After 15 min, phosphate buffer was replaced with basic buffer to stop engulfment and quench extracellular bacteria fluorescence for imaging.

To visualize the bead and bacteria engulfment by *Dictyostelium* cells, vegetative or developed *WT* and mutants expressing different protein markers were harvested, washed with PB, and settled in a 4-well chamber for 10 min. A 10-fold excess of beads was added to the *Dictyostelium* cells, and the engulfment process was recorded with a Zeiss LSM 880 Laser Scanning Microscope with a 60×; 1.3 NA Plan-Neofluar objective lens. To quantify LPS-coated bead engulfment, cells were incubated with LPS beads for 5 min and recorded. The number of beads engulfed by each cell was counted. For each cell line, about 40 cells were included for quantification.

### LPS binding

*WT* and *far1*^*−*^ cells were incubated with 10 μg/ml FITC-labeled LPS purified (*E*. *coli* O111:B4, Sigma) for 15 min at 4°C in the absence or presence of 1 mM folic acid. Binding was determined by flow cytometry. The representative result was shown, and quantitation results from 3 independent repeats were presented as mean fluorescence intensity (MFI) in Fig 1.

### fAR1-ΔN mutant protein generation

fAR1-ΔN-YFP were generated by inverse PCR in which pDV-fAR1-YFP was used as the template. The first 29 amino acids were kept as the signal peptide. Amino acids from position 30 to 355 of fAR1 encoding sequence were deleted. *far1*^*−*^ cells were transfected, and a population was selected by growing them in D3-T medium containing 50 μg/ml G418. Constructs were confirmed by DNA sequencing.

### Computational simulation

The computational framework of Neilson and colleagues [32,62] simulates migration using a slightly modified version of the Meinhardt mathematical model [63], in which an “activator” drives the movement of the cell perimeter in the outward-normal direction. Considering their simplicity, these simulations yield remarkably plausible biological behavior. However, if the cell contacts another object, the system generates stereotypical behavior that blocks further movement. To permit migration in the presence of obstacles such as bacteria, parts of the cell boundary that are found to lie inside an obstacle are projected (in the inward-normal direction) onto the boundary of the obstacle. To prevent the simulated cell from immobilizing upon contact with an obstacle, the activator is then quenched at any points of contact (simulating the equivalent of the “stall force” of normal actin), allowing the edges of the pseudopod to continue. We defined full engulfment as when the simulated cell boundary wraps around an obstacle and makes contact with itself, and we added a rule so that, following engulfment, the obstacle is removed, and the cell boundary joined together at the outermost points of contact. Chemoattractants behave exactly as in the previous work [32], except they are tied to the surface of the particle instead of diffusible. By modeling the obstacles in this fashion, the local attractant excites the activator at points of the cell boundary that are very close to (but not quite touching) the obstacle, causing the cell to wrap itself around the obstacle and allowing the simulated cell to phagocytose obstacles that are otherwise too large to engulf.

### Structural alignment, modeling, and docking

To explore how fAR1 recognizes ligands, we first analyzed amino acid sequence of fAR1, using Protter program [64], and found that it contains 1 extracellular N-terminal domain, 7 transmembrane domains, and 1 intracellular C-terminal domain (Fig 1A). Then, we input the sequence of the extracellular domain into an HHpred program [65] to align with the protein structures available in Protein Data Bank (PDB) by default parameter setting. All top-20 hits are bacterial proteins whose crystal structures fold as VFT structures. We then submitted the extracellular sequence of fAR1 to the online I-TASSER server [66], using all default settings to generate the homology model, which was used for docking. The three-dimensional conformer of folic acid (Pubchem CID 6037) was downloaded from PubChem 3D [67], which was used as the starting conformation for docking. Flexible docking of folic acid into the fAR1 model was performed by the program Glide in the Maestro suite from Schrodinger (v. 2016–2), using the induced fit docking protocol [68]. Docking was accomplished using the Extra Precision (XP) Glide [69]; all other parameters were defaults. A folic acid molecule gave Glide docking scores corresponding to nanomolar dissociation constant (Kd). Figures of the docked poses were prepared using Chimera 1.12 [70]. The pose with the highest docking score of folic acid is shown in Fig 1A.

### Statistics

The statistical significance was assessed using analysis of variance with the two-tailed unpaired Student *t* test. Data are presented as mean ± SD unless stated otherwise.

## Supporting information

S1 Fig LPS triggers chemotactic signaling through fAR1 and G proteins, related to Fig 1. (A-C) Vegetative *WT* and mutant cells expressing RBD-GFP (A), PH_CRAC_-GFP (B), and LimEΔcoil-GFP (C) were stimulated with LPS at 0 s. The transient increase in fluorescence intensity was measured at the plasma membrane and graphed. The intensity of the GFP signal was normalized to the first frame of each set of cells. Mean and SD of 10 cells from *gα2*^−^ and *gα4*^−^ are shown for the time course in (D). Scale bar: 2 μm. GFP, green fluorescent protein; LimEΔcoil, partial sequences of LimE protein; LPS, lipopolysaccharide; PH_CRAC_, PH domain of cytosolic regulator of adenylyl cyclase; RBD, Ras binding domain; WT, wild-type
(TIF)

S2 Fig VFT domain of fAR1 responses to saccharide region of LPS, related to Fig 1. (A) Construction of fAR1ΔN mutants. The truncation part of VFT is highlighted in a light gray box. (B) fAR1-Y/*far1*^*−*^ and fAR1ΔN-Y/*far1*^*−*^ cells were visualized by confocal microscopy. Scale bar: 5 μm. (C-E) Vegetative *WT* and mutant cells expressing LimEΔcoil-GFP were stimulated with Rd-LPS (C), Rc-LPS (D), or Ra-LPS (E) at 0 s. The transient increase in fluorescence intensity was measured at the plasma membrane and graphed. The intensity of the GFP signal was normalized to the first frame of each set of cells. Mean and SD from 10 cells are shown for the time course. Scale bar: 2 μm. fAR1, folic acid receptor 1; LPS, lipopolysaccharide; VFT, Venus-Flytrap.
(TIF)

S3 Fig Immobile chemoattractants promote particle engulfment, related to Fig 2. (A) Simulation of cell engulfment of circular obstacle with variable size (radius) coated with increasing amount of adhesion molecules or chemoattractants. Phagocytosis efficiency increases when chemoattractant concentration increases but not when adhesive molecule concentration increases; phagocytosis efficiency decreases when the target size (radius) increases. (B) Developed *D*. *discoideum WT*, *gα4*^*−*^, and *far1*^*−*^ cells expressing LimEΔcoil-GFP were incubated with cAMP-coated beads. The beads triggered phagocytic cup formation and engulfment. Scale bar: 2 μm. (C) Developed *D*. *discoideum gβ*^*−*^, *gα2*^*−*^, and *car1*^*−*^ cells expressing LimEΔcoil-GFP were incubated with cAMP-coated beads. The beads failed to trigger phagocytic cup formation and engulfment. Scale bar: 2 μm. (D) IL-8 coated on the bead surface promotes phagocytic cup formation in HL60 cells. Phagocytosis of IL-8-coated beads by human HL60 cells expressing actin-mCherry (red). Scale bar, 5 μm. E. Uncoated beads failed to trigger phagocytic cup formation in human HL60 cells. Scale bar: 5 μm. F. IL-8 coated beads engulfment by human HL60 cells were inhibited by pertussis toxin. Scale bar: 5 μm. IL-8, interleukin 8.
(TIF)

S4 Fig LPS-induced chemotaxis is dependent on fAR1 and G proteins, related to Fig 3. EZ-TAXIScan chemotaxis toward a linear LPS gradient of vegetative *WT*, *far1*^*−*^, *gβ*^*−*^, and fAR-Y/*far1*^*−*^ cells. Images were recorded every 15 s. A linear gradient of LPS in the channel formed from bottom to top in the figure. Images of each cell line at time 0, 20, and 40 min are shown. fAR1, folic acid receptor 1; LPS, lipopolysaccharide; WT, wild-type.
(TIF)

S1 Video Immobilized chemoattractant on particle surface can promote engulfment, related to Fig 2. Top left, simulated cell migration through pseudopod formation (green). Top right, simulated cell migration in the presence of a circular obstacle without any coating. Bottom left, simulation of cell migration in the presence of circular obstacle coated with adhesion molecules. Bottom right, simulated cell migration in the presence of a circular obstacle coated with chemoattractant on surface.
(AVI)

S2 Video LPS triggers engulfment through fAR1 and Gβ, related to Fig 3. Vegetative *WT* and mutant cells expressing LimEΔcoil-GFP or PH_CRAC_-GFP were incubated with LPS-coated beads and monitored by confocal microscopy. Top left, LimEΔcoil-GFP/*WT;* Top right, PH-GFP/*WT*; Middle left, LimEΔcoil-GFP/*far1*^*−*^; Middle right, PH-GFP/*far1*^*−*^; Bottom left, LimEΔcoil-GFP/*gβ*^*−*^; Bottom right, PH-GFP/*gβ*^*−*^. fAR1, folic acid receptor 1; LimEΔcoil, partial sequences of LimE protein; LPS, lipopolysaccharide; PH_CRAC_, PH domain of cytosolic regulator of adenylyl cyclase; WT, wild-type.
(AVI)

S1 Data Raw numerical data. All individual data that underlie the data summarized shown in the figures throughout the manuscript are shown and organized by tabs for each figure panel.
(XLSX)

## Abbreviations

BAI1: brain angiogenesis inhibitor I
cAR1: cAMP receptor 1
CaSR: Ca^2+^-sensing receptors
ERK2: extracellular signal-regulated kinase 2
fAR: folic acid receptor
FITC: fluorescein isothiocyanate
FSC: forward scatter
GABA_B_Rs: gamma-aminobutyric acid type B receptors
GPCR: G-protein-coupled receptor
IgG: immunogloblulin
IL-8: interleukin 8
LimEΔ: partial sequences of LimE protein
LPS: lipopolysaccharide
MAMP: microbial-associated molecular pattern
MFI: mean fluorescence intensity
mGlur: glutamate receptor
PBP: periplasmic acid-binding protein
PH_CRAC_: PH domain of cytosolic regulator of adenylyl cyclase
PI3K: phosphatidylinositol-4,5-bisphosphate 3-kinase
PIP3: phosphatidylinositol (3,4,5)-trisphosphate
PRR: pattern-recognition receptor
ROI: region of interest
SibA: Similar to Integrin Beta A
SSC: side scatter
T1R: taste receptor
TLR: Toll-like receptor
V2R: pheromone receptor
VFT: Venus-Flytrap
WT: wild type
