# Supplementary figures and images for: A G-protein-coupled chemoattractant receptor recognizes lipopolysaccharide for bacterial phagocytosis
Source: PLoS Biol. 2018 May 25;16(5):e2005754. doi: 10.1371/journal.pbio.2005754 (PMC5969738; doi:10.1371/journal.pbio.2005754)

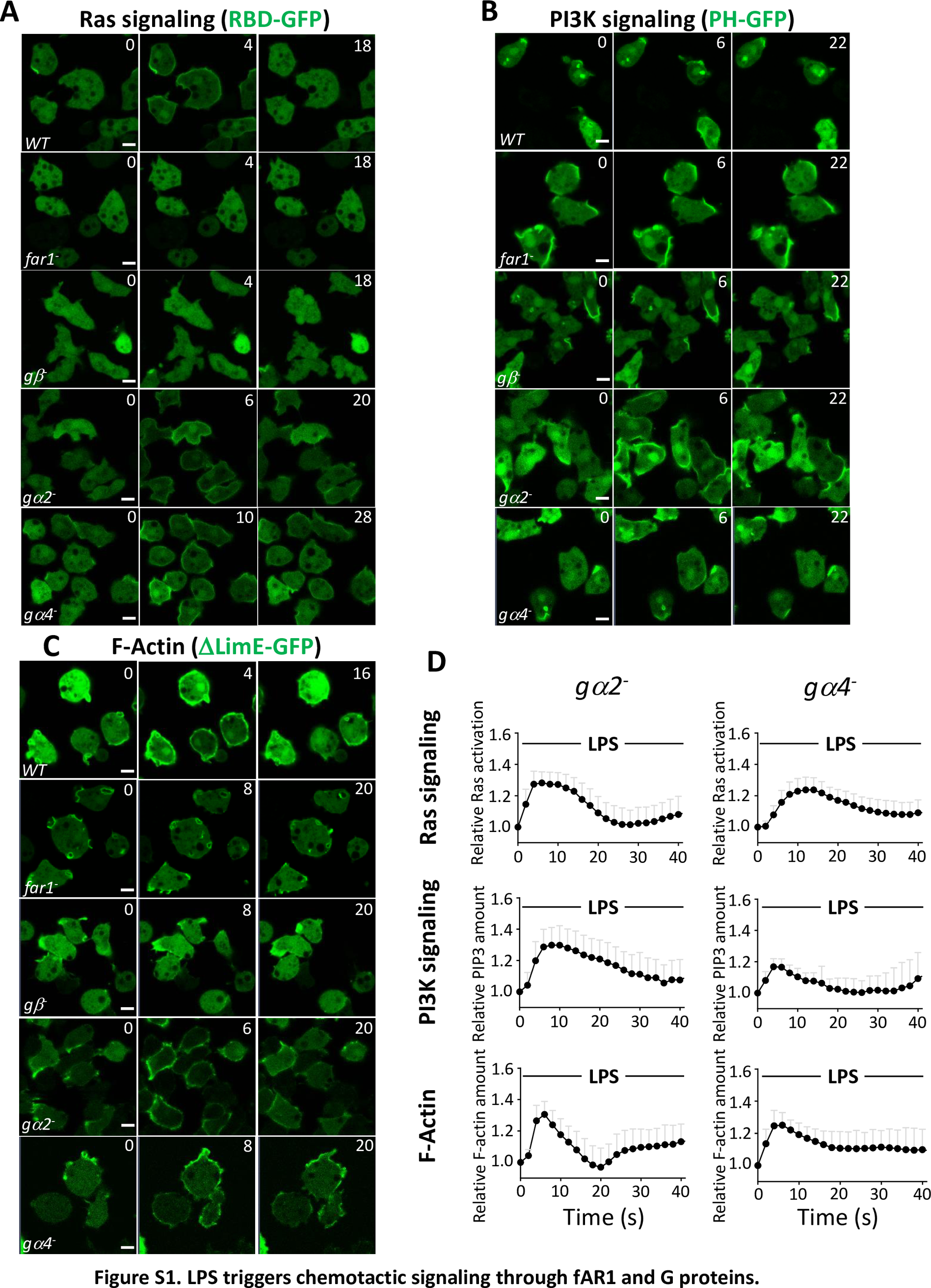

Supplement: S1 Fig — (A-C) Vegetative WT and mutant cells expressing RBD-GFP (A), PHCRAC-GFP (B), and LimEΔcoil-GFP (C) were stimulated with LPS at 0 s. The transient increase in fluorescence intensity was measured at the plasma membrane and graphed. The intensity of the GFP signal was normalized to the first frame of each set of cells. Mean and SD of 10 cells from gα2− and gα4− are shown for the time course in (D). Scale bar: 2 μm. GFP, green fluorescent protein; LimEΔcoil, partial sequences of LimE protein; LPS, lipopolysaccharide; PHCRAC, PH domain of cytosolic regulator of adenylyl cyclase; RBD, Ras binding domain; WT, wild-type (TIF) [file pbio.2005754.s001.tif]

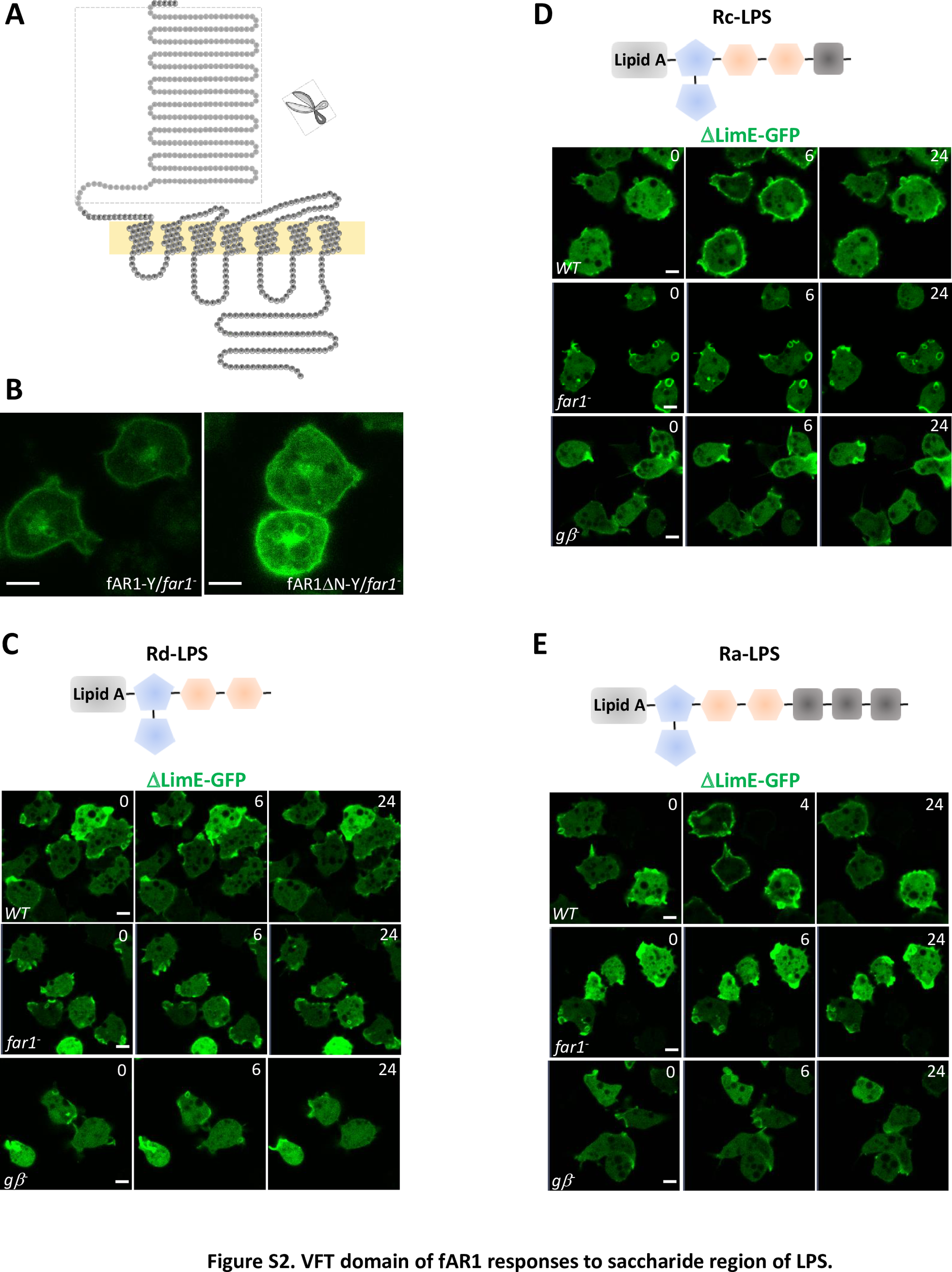

Supplement: S2 Fig — (A) Construction of fAR1ΔN mutants. The truncation part of VFT is highlighted in a light gray box. (B) fAR1-Y/far1− and fAR1ΔN-Y/far1− cells were visualized by confocal microscopy. Scale bar: 5 μm. (C-E) Vegetative WT and mutant cells expressing LimEΔcoil-GFP were stimulated with Rd-LPS (C), Rc-LPS (D), or Ra-LPS (E) at 0 s. The transient increase in fluorescence intensity was measured at the plasma membrane and graphed. The intensity of the GFP signal was normalized to the first frame of each set of cells. Mean and SD from 10 cells are shown for the time course. Scale bar: 2 μm. fAR1, folic acid receptor 1; LPS, lipopolysaccharide; VFT, Venus-Flytrap. (TIF) [file pbio.2005754.s002.tif]

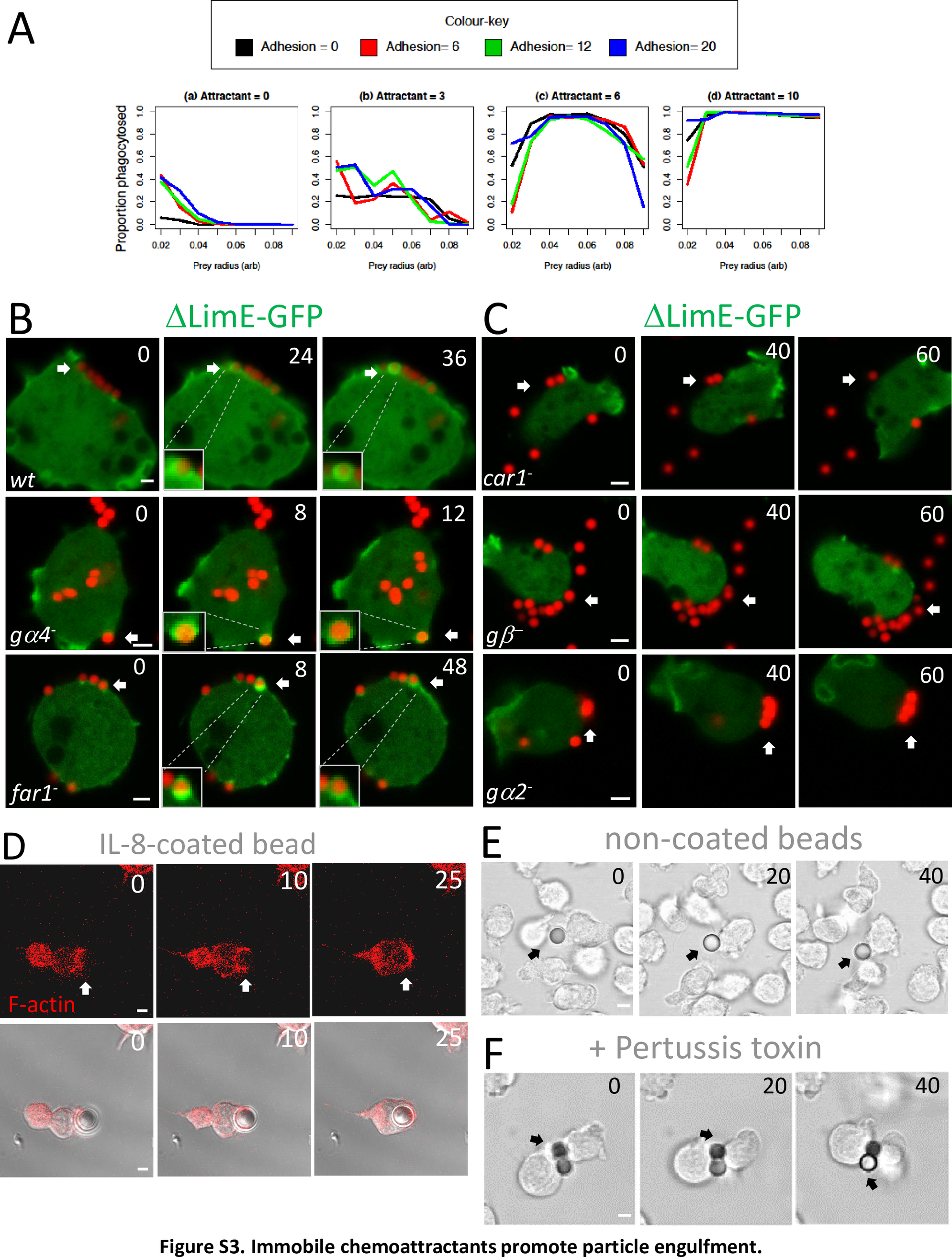

Supplement: S3 Fig — (A) Simulation of cell engulfment of circular obstacle with variable size (radius) coated with increasing amount of adhesion molecules or chemoattractants. Phagocytosis efficiency increases when chemoattractant concentration increases but not when adhesive molecule concentration increases; phagocytosis efficiency decreases when the target size (radius) increases. (B) Developed D. discoideum WT, gα4−, and far1− cells expressing LimEΔcoil-GFP were incubated with cAMP-coated beads. The beads triggered phagocytic cup formation and engulfment. Scale bar: 2 μm. (C) Developed D. discoideum gβ−, gα2−, and car1− cells expressing LimEΔcoil-GFP were incubated with cAMP-coated beads. The beads failed to trigger phagocytic cup formation and engulfment. Scale bar: 2 μm. (D) IL-8 coated on the bead surface promotes phagocytic cup formation in HL60 cells. Phagocytosis of IL-8-coated beads by human HL60 cells expressing actin-mCherry (red). Scale bar, 5 μm. E. Uncoated beads failed to trigger phagocytic cup formation in human HL60 cells. Scale bar: 5 μm. F. IL-8 coated beads engulfment by human HL60 cells were inhibited by pertussis toxin. Scale bar: 5 μm. IL-8, interleukin 8. (TIF) [file pbio.2005754.s003.tif]

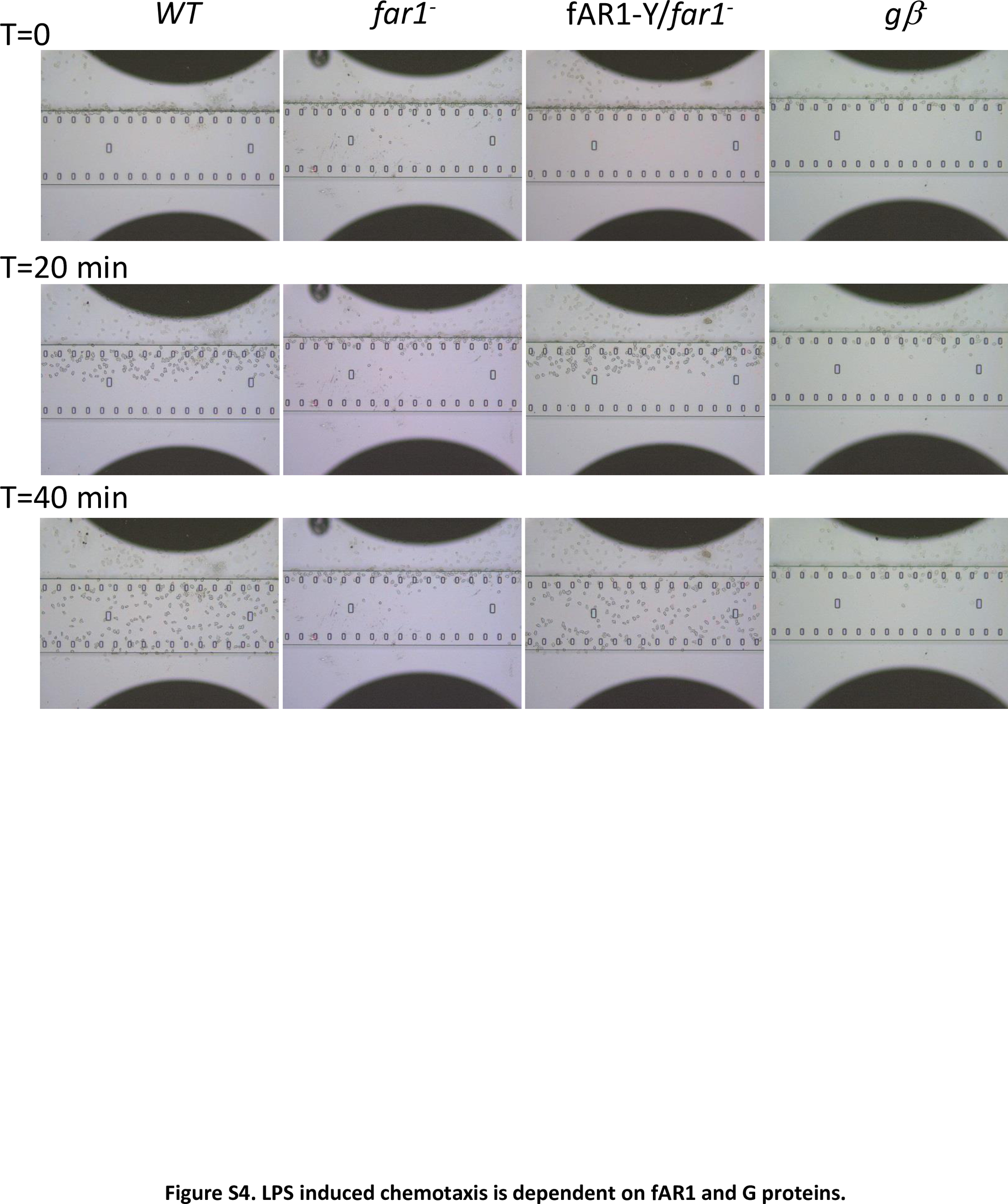

Supplement: S4 Fig — EZ-TAXIScan chemotaxis toward a linear LPS gradient of vegetative WT, far1−, gβ−, and fAR-Y/far1− cells. Images were recorded every 15 s. A linear gradient of LPS in the channel formed from bottom to top in the figure. Images of each cell line at time 0, 20, and 40 min are shown. fAR1, folic acid receptor 1; LPS, lipopolysaccharide; WT, wild-type. (TIF) [file pbio.2005754.s004.tif]
